# Notch Lineages and Activity in Intestinal Stem Cells Determined by a New Set of Knock-In Mice

**DOI:** 10.1371/journal.pone.0025785

**Published:** 2011-10-03

**Authors:** Silvia Fre, Edouard Hannezo, Sanja Sale, Mathilde Huyghe, Daniel Lafkas, Holger Kissel, Angeliki Louvi, Jeffrey Greve, Daniel Louvard, Spyros Artavanis-Tsakonas

**Affiliations:** 1 Morphogenesis and Intracellular Signaling, Institut Curie-UMR144 CNRS, Paris, France; 2 Physical Chemistry Curie, Institut Curie-UMR 168 CNRS-UPMC, Paris, France; 3 Department of Cell Biology, Harvard Medical School, Boston, Massachusetts, United States of America; 4 Department of Developmental Biology and Genetics, Institut Curie-UMR3215 CNRS-U934 Inserm, Paris, France; 5 TaconicArtemis GmbH, Köln, Germany; 6 Program on Neurogenetics and Departments of Neurosurgery and Neurobiology, Yale School of Medicine, New Haven, Connecticut, United States of America; 7 Exelixis, Inc., South San Francisco, California, United States of America; 8 Collège de France, Paris, France; National Institutes of Health (NIH), United States of America

## Abstract

The conserved role of Notch signaling in controlling intestinal cell fate specification and homeostasis has been extensively studied. Nevertheless, the precise identity of the cells in which Notch signaling is active and the role of different Notch receptor paralogues in the intestine remain ambiguous, due to the lack of reliable tools to investigate Notch expression and function *in vivo*. We generated a new series of transgenic mice that allowed us, by lineage analysis, to formally prove that Notch1 and Notch2 are specifically expressed in crypt stem cells. In addition, a novel Notch reporter mouse, Hes1-EmGFP^SAT^, demonstrated exclusive Notch activity in crypt stem cells and absorptive progenitors. This roster of knock-in and reporter mice represents a valuable resource to functionally explore the Notch pathway *in vivo* in virtually all tissues.

## Introduction

Extensive work on the mouse intestine established the existence of a multipotent cell population that has the capacity to regenerate throughout life and is responsible for the rapid and continuous renewal of the intestinal epithelium [1]. Through the identification of specific cellular markers, two stem cell populations have been identified in distinct positions within the intestinal crypts. Lgr5 positive stem cells, also referred to as crypt base columnar (CBC) cells, dwell at the bottom of the crypt, intercalated with differentiated Paneth cells in the small intestine [2]; Bmi1 positive stem cells are predominantly located just above the Paneth cell compartment and are referred to as “+4 cells”, indicating their position from the bottom of the crypt [3]. Both stem cell populations are actively cycling and show similar developmental multipotency and long-term regeneration potential, but have distinct cell cycle requirements. CBC cells are extremely sensitive to the loss of CDC25 function, a cell cycle protein controlling cell proliferation, and respond by undergoing differentiation prematurely, while +4 cells remain insensitive [4]. The existence of a third kind of stem cells, slowly cycling or quiescent, has also been recently reported, but their biological properties have yet to be characterized [5]. It is thought that stem cells give rise to short-lived transit-amplifying progenitors, which undergo a limited number of divisions while apically migrating. These are still uncommitted crypt cells that will eventually give rise to the fully differentiated intestinal lineages.

The Notch signaling pathway is a major regulator of cell fate in metazoan development, linking the fate of one cell to that of a cellular neighbour. This is achieved through the interaction of membrane-bound ligands in one cell to the Notch surface receptor expressed on an adjacent cell. A wealth of studies implicated Notch signalling in controlling the fate of early precursors, indeed stem cells, in many different organs [6], [7], [8], so that it is now recognized as a major player in various aspects of stem cell biology, including their maintenance and differentiation [9]. In the intestine, we and others have shown that Notch signals are essential in maintaining tissue homeostasis and continuous renewal. While abolishing Notch signaling induces an arrest of crypt cell division and the conversion of crypt progenitors into secretory cells [10], [11], [12], constitutive activation of Notch1 leads to a dramatic increase in the number of proliferating cells in the intestinal epithelium, accompanied by impairment of differentiation [13].

Notwithstanding the clear implications of Notch signaling in intestinal stem cell homeostasis, it is unclear which of the four Notch receptor paralogues are active in intestinal stem cells and whether Notch expression marks multipotent stem cells, or lineage-restricted precursors. Indeed the specific role of Notch paralogues is an aspect of Notch biology that is not well understood and the reagents to rigorously address this question are lacking.

Here, we set out to formally assess the expression and activity of Notch receptors in intestinal stem cells *in vivo* using a roster of new transgenic mice we have generated, that allow fate mapping of specific cell lineages whose precursors express specific Notch receptor paralogues, as well as monitoring signaling activity using Notch reporter mice. The value of the transgenic strains we generated is demonstrated in this study for the intestine, where we show that both Notch1 and Notch2 receptors are specifically expressed in crypt stem cells. Using a Notch activity reporter mouse, we also ascertain that Notch signaling is active in intestinal stem cells, as well as in absorptive progenitors, while in cells destined to adopt a secretory fate, and in terminally differentiated cells, Notch activity is undetectable.

## Results

### Cre gene targeting for Notch paralogue-specific expression

In order to establish the identity of the cells expressing the different Notch receptors and eventually trace their lineage, we generated four transgenic lines in which the Cre-estrogen receptor binding domain fusion (Cre-ER) [14] is knocked-in into the first exon of each of the four Notch receptor paralogues (N1 through N4, henceforth referred to as N1-CreERT2^SAT^, N2-CreERT2^SAT^, N3-CreERT2^SAT^ and N4-CreERT2^SAT^). In each of the four lines, one allele of the targeted Notch locus expresses the tamoxifen inducible Cre-ER recombinase under the control of the respective Notch paralogue promoter, while the other allele remains functional (Figure S1A). All lines are viable and fertile as heterozygotes, whereas homozygous N1-CreERT2^SAT^ and N2-CreERT2^SAT^ mice die during embryogenesis, as described for their respective knock-outs [15], [16].

We validated the four N(1–4)-CreERT2^SAT^ knock-in lines by crossing them to a Rosa26 LacZ reporter strain (R26R) [17]. Adult N(1–4)-CreERT2^SAT^/+; R26R/+ and control N(1–4)-CreERT2^SAT^/+ mice were induced with a single dose (50 mg/kg of mouse body weight) of 4-Hydroxytamoxifen (4-OHT) and analyzed after 24 hours. Tissue samples from adult bi-genic and control mice were visualized for β-galactosidase expression in order to detect the cells that had undergone recombination. Our results in heart, liver and brain corroborate and extend previous reports for Notch receptor distribution in these tissues [18], [19], [20], [21], and indicate that the Cre recombinase is indeed expressed according to the expression pattern of the four Notch paralogues (Figure S1B–K).

### Notch receptor expression is associated with crypt stem cells

The ability to conditionally mark Notch-expressing cells allows us to follow the cell lineages associated with each Notch receptor paralogue expression and thus we used these transgenic tools to examine the expression of Notch receptors in the intestine and their relationship to the renewal of this tissue in adult mice.

**Figure 1:**
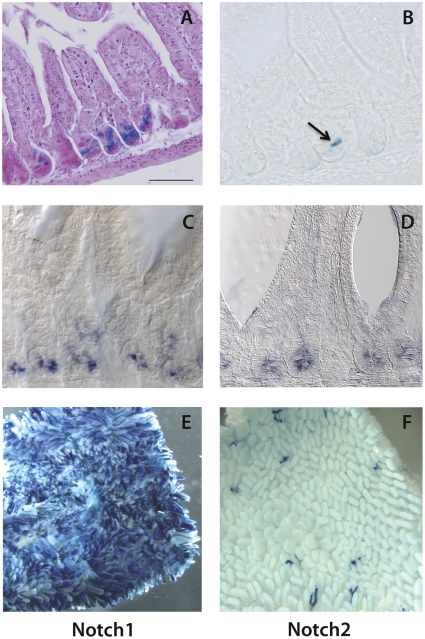
Notch1 and Notch2 expression in the small intestine. X-gal staining (blue) of thin sections of proximal small intestine of N1-CreERT2^SAT^/+;R26R/+ (A) and N2-CreERT2^SAT^/+;R26R/+ (B) 24 hours after tamoxifen administration shows that Notch1 and Notch2 are expressed only in the intestinal crypts. C–D) In situ hybridization for Notch1 (C) and Notch2 (D) demonstrates specific expression in intestinal crypts. E–F) Whole mounts duodenum of N1-CreERT2^SAT^/+;R26R/+ (E) and N2-CreERT2^SAT^/+;R26R/+ (F) 7 months after tamoxifen administration demonstrates long-term labeling of X-gal marked Notch lineages. Counterstain: hematoxylin/eosin in A. Scale bar: 50 µm in A–D and 1 mm in E–F.

Following induction of 4 week-old N(1–4)-CreERT2^SAT^/+;R26R/+ double transgenic mice with one pulse of tamoxifen (50 mg/kg of body weight), Notch1 and Notch2 mark exclusively epithelial crypt cells of the adult small intestine, when examined 12, 18 and 24 hours after tamoxifen administration (Figure 1A–B), in agreement with in situ hybridization results (Figure 1C–D). Conversely, Notch3 and Notch4 are not expressed in the gut epithelium but in the endothelial and mesenchymal cells surrounding it (data not shown). This analysis also revealed that far fewer cells express Notch2 than Notch1.

**Figure 2:**
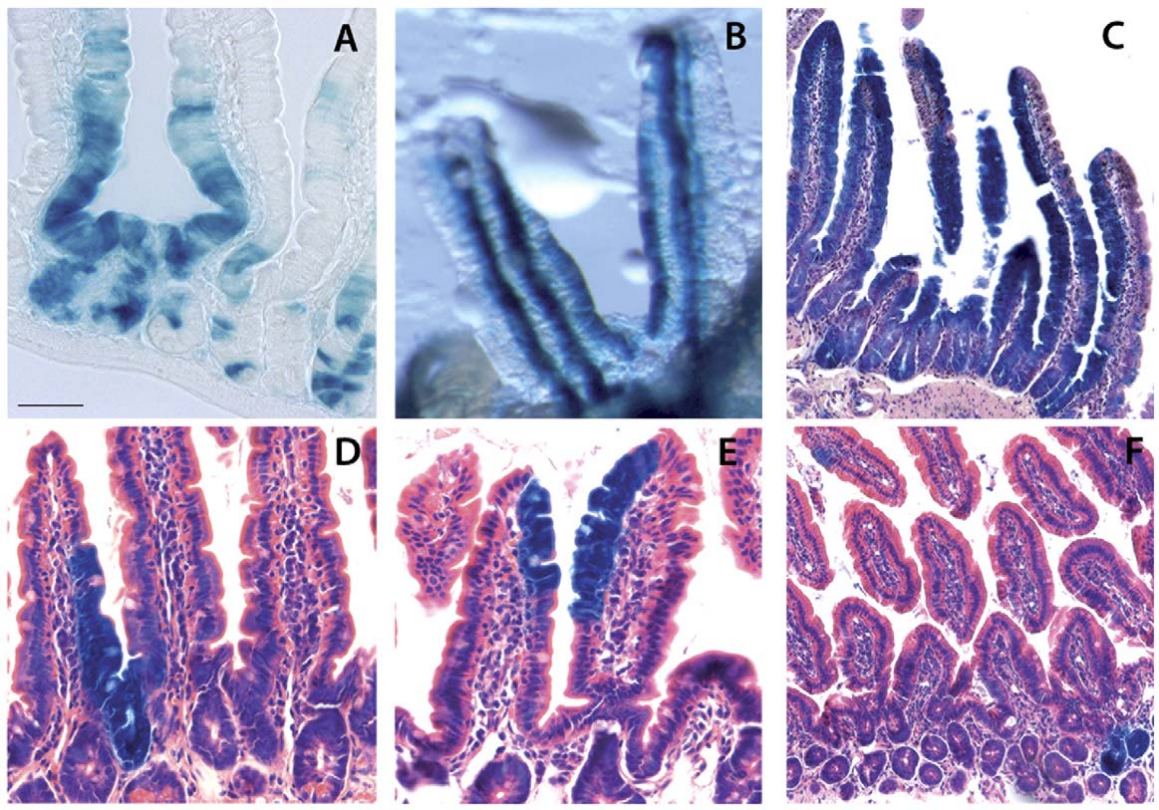
Tracing of Notch1 and Notch2 lineages shows stem cell labeling. Four week-old N1-CreERT2^SAT^/+;R26R/+ (A–C) and N2-CreERT2^SAT^/+;R26R/+ (D–F) mice were injected with tamoxifen and analyzed for β-galactosidase expression (blue) after 3 days (A,D), 7 days (B,E) and 60 days (C,F). The lineage of Notch1-expressing cells is shown in thin longitudinal sections in A and C and in whole mounts in B to better visualize continuous streams of labeled cells. Sections are counterstained with hematoxylin/eosin in C–F. Scale bar: 40 µm in A,D,E; 50 µm in B and 80 µm in C,F.

Three days after tamoxifen induction, we detected marked progeny of Notch-expressing cells outside the crypt, in differentiated villus cells (Figure 2A,D); labeled clones formed continuous streams of cells vertically aligned to the crypt-villus axis and reached the villus top by seven days (Figure 2B), consistent with the kinetics of cell replacement in the intestine. Importantly, β-galactosidase positive cells persisted for 30 days, 60 days (Figure 2C,F) and even 7 months after a single tamoxifen induction (Figure 1E–F), continuing to give rise to differentiated progeny, indicating labeling of long-lived intestinal stem cells endowed with self-renewal capacity. All experiments indicate that while both Notch1 and Notch2 mark stem cells, the incidence of Notch1-expressing cells is far greater (as clearly illustrated in whole mount pictures in Figure 1E–F).

### Identification of Notch lineages in the intestine

In order to corroborate our findings with the R26R reporter strain, we crossed N(1–2)-CreERT2^SAT^ mice to a recently developed Cre-sensitive double fluorescent reporter line, R26^mTmG^ [22]. In N(1–4)-CreERT2^SAT^/R26^mTmG^ mice, the membrane-associated tomato fluorescent protein (mT) is expressed in all cells until tamoxifen injection, after which membrane-associated GFP (mG) marks cells targeted by Cre recombination. The availability of the two strains offered the possibility to corroborate our results in two distinct genetic backgrounds: in the absence of tamoxifen treatment, reporter expression was not detected in both double transgenic mice, indicating absence of leakiness.

**Figure 3:**
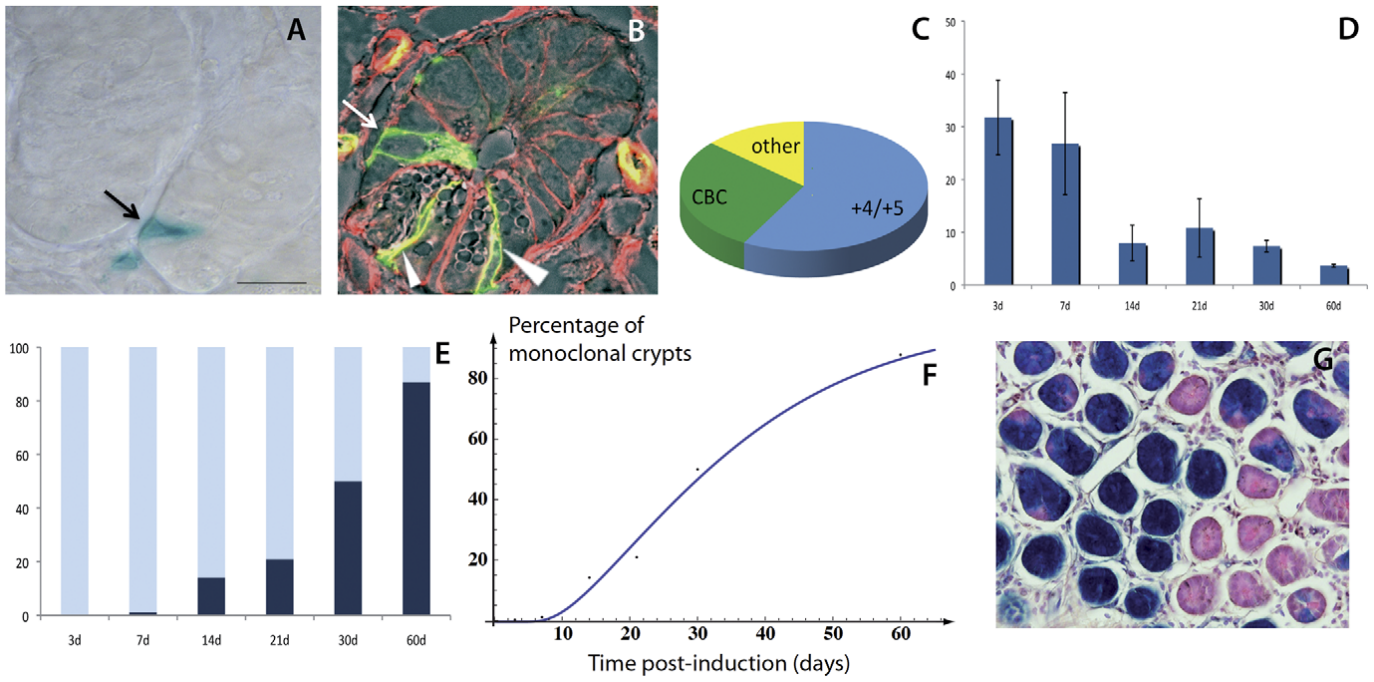
Kinetics of stem cell replacement and monoclonal conversion of Notch1-expressing cells. To define the localization of Notch1-expressing crypt cells, only crypts harboring a single marked cell were scored, as shown in A. Cells were classified as “CBCs” (marked by white arrowheads in B), “+4” (indicated by a white arrow in B) or “other” when it was not possible to clearly assign their position. In B, N1-CreERT2^SAT^/+;R26^mTmG^/+ mice were injected with tamoxifen at 4 weeks and analyzed 24 h after induction. Genetically marked cells show membrane-bound green fluorescence (GFP), while membrane-anchored Tomato (red) fluorescence is present in non-recombined cells. C: graphic view of the quantification of labeled cells. At least five fields from a single intestine were counted and at least three mice per time point (12 h, 18 h, 24 h) were scored independently by two researchers. The total number of crypts counted was 1095. D) Marked crypts were counted at different time points after tamoxifen injection (x axis). The Y axis represents the percentage of labeled crypts over the total crypt population. E) Graphic representation of the percentage of fully labeled (dark blue) or mosaic crypts (light blue) among the population of marked crypts. F) Data of the monoclonal conversion of marked crypts (dots), compared with a fit (straight line), applying the neutral drift theory [17]. The only fitting parameter is the stem cell renewal rate λ = 1.32±0.05/day. G) Representative cross section of 60 days chase showing fully labeled (all blue), partially labeled and non-recombined (pink) crypts used to quantitatively evaluate the monoclonal conversion kinetics. Counterstain: hematoxylin and eosin. Scale bar: 10 µm in A, B.

To score the location of the crypt cells expressing the two Notch receptors, we reduced the tamoxifen dose to 5 mg/kg of mouse body weight, which allowed us to theoretically label a single cell per crypt. At this low tamoxifen dose, approximately 30% of the total crypts harbor labeled cells. We proceeded counting crypts harboring a single marked cell (an example of a “scorable” crypt is shown in Figure 3A) and classified its localization as “CBCs” or “+4 cells”, as exemplified in Figure 3B. While, as expected from the above analysis, we very rarely succeeded in identifying Notch2 positive crypt cells, preventing rigorous statistical analysis, the position of the Notch2-positive cells identified could not be distinguished from the position of the Notch1-positive cells. As presented in the pie graph in Figure 3C, we find Notch1-expressing cells predominantly in the +4/+5 position, though approximately 30% of marked cells are also found as CBC cells intermingled among Paneth cells. By topological and morphological criteria alone, therefore, Notch1 appears to be expressed in both crypt stem cell sub-populations.

**Figure 4:**
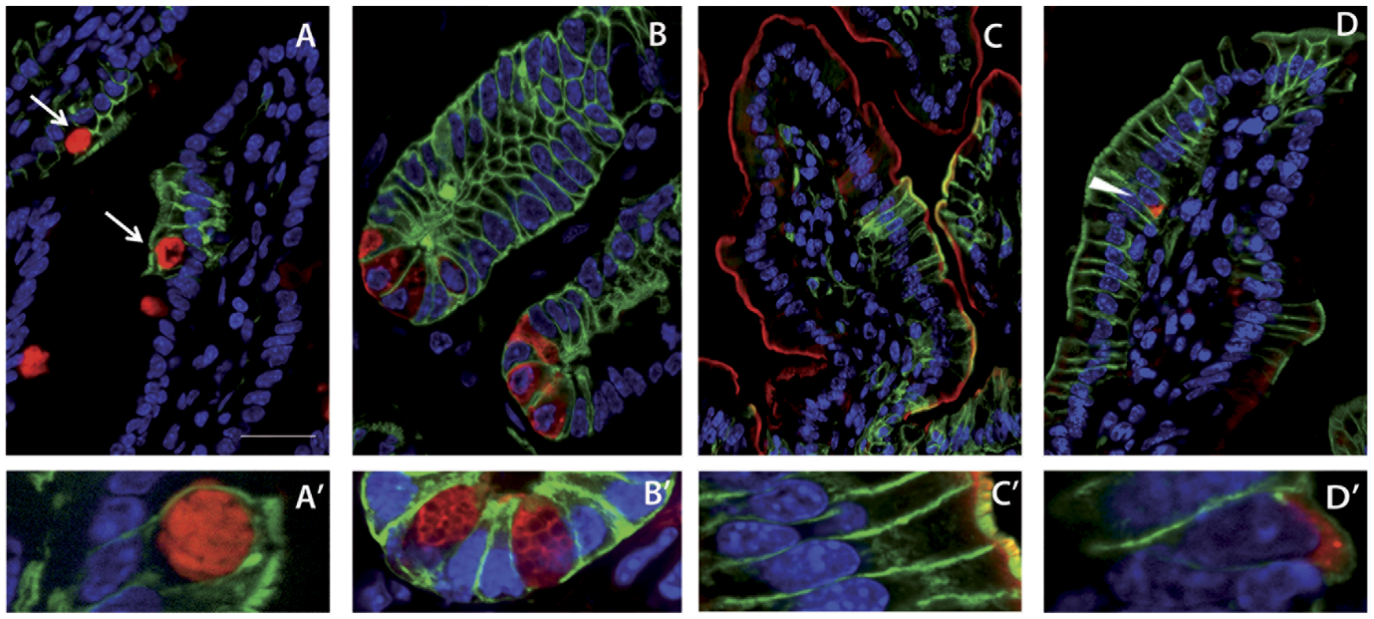
Notch1-expressing cells in the intestine are multipotent. N1-CreERT2^SAT^/+;R26^mTmG^/+ mice were analyzed 30 days after tamoxifen administration. Notch1-expressing crypt cells give rise to all four differentiated cell types of the intestine, as illustrated by the concomitant labeling of membrane-tagged GFP (Notch progeny in green) and A-A′) UEA1 staining (red) marking Goblet cells indicated by the arrows; B-B′) anti-lysozyme staining (red) recognizing Paneth cells; C-C′) anti-villin staining (red) marking the apical brush border of enterocytes; and D-D′) anti-chromogranin immuno-labeling (red) revealing the position of enteroendocrine cells (indicated by a white arrowhead in D). A′–D′ show higher magnifications of the four cell types presenting both membrane-tethered green and red fluorescence. Nuclei are marked in blue with DAPI. Scale bar: 50 µm in A–D; 10 µm in B′ and 5 µm in A′, C′ and D′.

Mapping the fate of Notch1-expressing crypt cells revealed that Notch1 is indeed expressed in multipotent cells able to generate all four differentiated cell types of the intestine (Figure 4). The extreme longevity of Notch1-expressing cells in a tissue where almost complete cell renewal is normally achieved in a week, along with their multipotency, has allowed us to confirm that Notch1 is expressed *in vivo* in long lasting intestinal stem cells.

The analysis of the Notch1 lineage in the different tracts of the intestinal tube, shows a striking proximal to distal descending gradient in the small and large intestine, with highly abundant Notch1 lineage in the proximal duodenum and fewer labeled cells in the distal parts of the ileum and in the colon (Figure S2), as previously reported [10], [23], [24]. This spatial gradient probably reflects regional differences in Notch1 expression in the intestine, but it may also result from a lower efficiency in stem cell labeling in the distal bowel due to a slower division rate of colonic stem cells [2] or to decreased tamoxifen availability in the colon.

### Clonal dynamics of Notch 1 expressing stem cells

If indeed the Notch receptor marks genuine stem cells, we expected Notch cell lineages to show a dynamic profile compatible with the reported behavior of intestinal stem cells [25], [26]. Examining the populations kinetics of Notch1-expressing cells at different time points after Cre induction showed that the percentage of marked crypts gradually decreases over time when we used a diluted dose of tamoxifen (5 mg/kg of mouse body weight), eventually stabilizing around 2% within 60 days (see graph in Figure 3D).

It has been calculated that each crypt contains 14 to 16 stem cells [26], but only one of them generates the entire crypt progeny, a phenomenon termed monoclonal conversion [27], [28], [29]. Wich stem cell will eventually clonally repopulate the crypt is stochastic event [25], [26] founded on neutral competition between equipotent stem cells. Marking a single Notch-expressing stem cell in a crypt, reflects then 1/16 (6%) chance of labeling the cell that will generate the entire crypt, assuming that there are 16 stem cells per crypt. Given that 30% of all crypts are marked, we anticipated that only 6% of those would eventually give rise to fully labeled crypts during monoclonal conversion. Our finding therefore of a gradual decrease in the number of Notch marked crypt cells over time with a plateau of about 2% (Figure 3D) is in good agreement with what we would expect if the Notch receptor was expressed in the crypt stem cells.

When we examined the dynamics associated with the monoclonal conversion of marked crypts in relation to time after tamoxifen induction, we found a progressive increase in the number of fully labeled crypts as the chase time increases, reaching about 90% by 60 days (Figure 3E–G). Such behavior is expected from previous reports examining stem cell lineages and can be satisfactorily described by the neutral drift equations utilized broadly in population genetics [30]. We found that the stem cell replacement rate lambda is equal to 1.32/day, a value consistent with previous findings in the crypt [25], [26]. This analysis clearly associates Notch expression with long-lived intestinal stem cells.

### Notch signaling is active in crypt stem cells and is permissive for enterocyte differentiation

**Figure 5:**
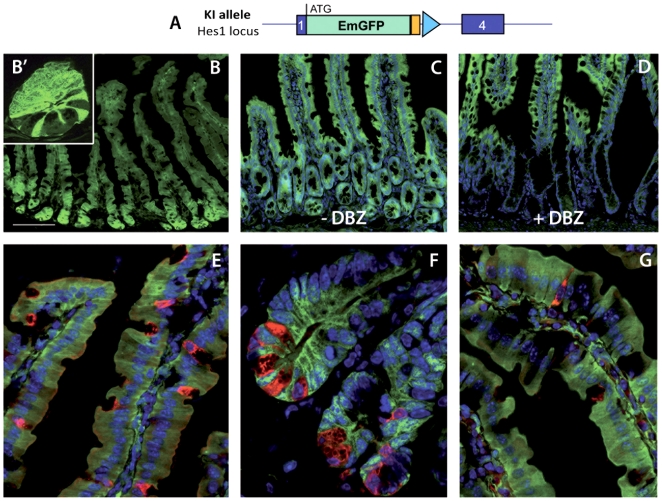
Expression of the Notch transcriptional target Hes1 in the intestine. A) Schematic representation of the targeted Hes1 locus in the Hes1-EmGFP^SAT^ reporter line. Hes1-EmGFP^SAT^ mice were analyzed for GFP expression in frozen sections (B–G). DBZ-treated animals (D) show absence of Hes1-EmGFP expression in crypt cells, while the signal in the villus compartment is unaltered by Notch inhibition, as compared to untreated mice (B and C). Immunofluorescent labeling of secretory cells reveals the complete absence of GFP label from differentiated secretory cells, detected by anti-mucin antibodies for Goblet cells (red) in E, anti-lysozyme staining for Paneth cells (red) in F (see also the absence of green fluorescence in Paneth cells in the inset B′) and anti-chromogranin staining for enteroendocrine cells and (red) in G. Nuclei are marked in blue with DAPI in C–G. Scale bar: 100 µm in B; 10 µm in B′; 80 µm in C–D and 40 µm in E–G.

Given that expression of the Notch receptor cannot be equated to Notch signal activation, it was important to assess whether cell populations that express Notch receptors actually signal. To this end, we generated and used a novel Hes1-EmGFP^SAT^ reporter mouse, in order to monitor Notch activity *in vivo* at a single cell level. In these mice, the expression of emerald GFP is under the control of the endogenous promoter of the Notch target gene Hes1 (Figure 5A). Analysis of the adult intestine of Hes1-EmGFP^SAT^ mice showed that undifferentiated crypt cells, including stem cells and progenitors, express high levels of Hes1 (Figure 5B), while GFP fluorescence is completely absent from all secretory cell types: Paneth, enteroendocrine and Goblet cells (Figure 5E–G). Some GFP expression is also detected in differentiated enterocytes in the villus compartment, although at significantly lower levels compared to crypt cells (see Figure 5B). This could result from persistence of GFP protein due to high protein stability, possibly suggesting that residual Notch activity is present in progenitor cells adopting an absorptive fate, or it may indicate that other pathways may activate the Hes1 promoter in enterocytes. In an attempt to examine if the enterocyte fluorescence reflects active Notch signaling, we then treated Hes1-EmGFP^SAT^ mice with the pharmacological Notch inhibitor dibenzazepine (DBZ) and were able to show that the residual GFP signal observed in the villi does not depend on active Notch signaling. Indeed, while GFP fluorescence in the crypts is completely abolished in DBZ-treated mice, detectable levels in the villi are unchanged by Notch inhibition (Figure 5C–D). These results are consistent with the absence of active Notch signaling in terminally differentiated villus cells. Given that the Hes1 protein is not detected in the villus compartment [12], [13], [31], [32], we conclude that the observed residual GFP signal is due to persistence of the GFP protein in the progeny of absorptive progenitor cells.

## Discussion

The generation of a new set of Notch transgenic mice allowed us a dissection of cell lineages associated with Notch receptor expression and activity in the intestine in an unprecedented manner. The data we obtained indicate that the Cre recombinase inserted into the first exon of each of the four Notch receptor paralogues is expressed in a pattern faithfully reflecting the endogenous expression of each receptor. While the gamut of tissues examined thus far is limited, the detailed characterization of the expression pattern in the intestine at the single cell level indicates that these reagents can be widely and ubiquitously used to examine Notch expression and, most importantly, allow the analysis of Notch lineages. Moreover, the Hes1 reporter we generated seems to accurately report Notch signaling activity in the intestine. We thus anticipate that the collection of transgenic reagents we generated will be broadly useful.

The importance of Notch signaling in intestinal renewal and differentiation, as well as in affecting tumor development in this tissue, is exemplified by the vast number of reports and reviews on this topic that appeared within the last few years (i.e. [10], [11], [12], [13], [24], [31], [33], [34], [35], [36]). Our results formally establish the specific expression of Notch1 and Notch2 in intestinal stem cells *in vivo* and ascertain that the pathway is indeed functional in these cells. While it is clear that Notch1 labeling is far more prominent than Notch2, the limitations of the Cre-mediated recombination method of marking cells do not allow us to determine if this difference is due to the relative expression levels of Notch1 and Notch2 or whether the two receptors mark distinct stem cell populations. Notch1 and Notch2 have been previously found to have redundant functions in the intestine, using conditional knockout mice for either one of the two receptors [11]. On the other hand, Wu et al. recently reported that the specific inhibition of Notch1, but not Notch2, by antagonistic antibodies is sufficient to reveal a partial Notch loss of function phenotype in the intestinal crypt, characterized by a decrease of proliferative cells and an increase of secretory goblet cells [37]. Our expression data show that while Notch1 expression is predominant in the proximal small intestine (Figure S2), Notch2 marks more efficiently cells in the distal tracts (data not shown). The regional differences we observe could therefore account for an overlapping, yet not completely redundant function of the two Notch receptors in the intestinal epithelium. This would be of importance as it is widely assumed that the specificity of the Notch1 and Notch2 receptors are equivalent, albeit displaying non-identical “pharmacologies”. Given the extraordinary sensitivity of cells to the dosage of Notch signaling activity [38], [39], subtle differences in the expression profiles of these two receptors may translate into distinct and developmentally significant signaling levels.

Activation of Notch signaling in intestinal stem cells has recently been described using a Notch1 activation-dependent knock-in mouse line, NIP1::CreERT2 [10]. In this study, stem cell labeling has been indirectly assessed, by analyzing the progeny of cells in which Notch was activated, eight months after tamoxifen administration. When we assessed Notch transcriptional activity *in vivo* at the time of dissection, using the Hes1-EmGFP^SAT^ reporter line, we observed GFP label in stem cells, but also in villus enterocytes, albeit at a lower intensity than in crypt cells. These observations are consistent with our previous findings showing that constitutively active Notch in adult mice blocks secretory cell differentiation, but it is permissive for the absortive cell fate [12], [31], [34]. We can thus extrapolate that Notch signaling is indeed active in crypt stem and possibly enterocyte progenitor cells.

The absence of Cre recombination in villus enterocytes in N1-CreERT2 mice may be explained by very low activity of the Notch1 promoter in these cells, not sufficient to reach the threshold for Cre-mediated recombination. Indeed, Kopinke and colleagues recently reported the expression of Hes1 in the intestinal crypt using a Hes1-CreERT2 mouse and found that Hes1 marks single cells at the crypt base or at the crypt-villus junction, but observed no label in the villus compartment [24]. Taken together, these combined results suggest that high levels of Notch signals are operating in crypt stem cells, whereas low signaling levels, below the Cre activation threshold, may be present in progenitor cells and may be important to funnel these cells to the enterocyte fate. Residual GFP label found in differentiated enterocytes in the villus may be due to persistence and dilution of GFP upon cell division of absortive progenitors due to high protein stability, as suggested by the fact that villus GFP is not changed by blockage of Notch activity in vivo.

Even if it is clear that Notch1 marks intestinal stem cells, the possibility that it may also be expressed in short-lived progenitor cells warrants some consideration. When we analyzed clonal dynamics, we observed that the percentage of marked crypts does not monotonously decrease during the first week, as the neutral drift theory would predict (no significant change between 3 days and 7 days in Figure 3D). If crypts with one labeled stem cell also contained some marked short-lived progenitors, the crypts where marked stem cells are replaced by neutral competition with unlabeled stem cells would still appear blue in the first week, even though they will eventually be monoclonally converted to white crypts. This behavior prompts us to consider the possibility that Notch1 may also mark short-lived progenitor cells, an option that would be compatible with the estimated progenitor renewal rate, suggested to be approximately 7 days [33]. In this context, it is worth noting that the insertion of the CreERT2 cassette in the Notch1–4 loci creates a null allele. Even if Notch1 and Notch2 heterozygous mice do not display any appreciable differences from wild type animals, the possibility that crypt stem cells carrying one functional Notch allele may behave slightly differently than wild type cells cannot be rigorously ruled out on the basis of our analysis.

This study indicates that Notch signaling is required to maintain multipotent stem cells in the intestine, while our previous studies established that Notch activation might affect the proliferative potential of stem cells [13]. These results emphasize the central role of Notch in the maintenance of intestinal stem cells and are compatible with our findings that the synergy between Notch activation and other oncogenic stimuli is involved in the initiation of neoplastic conditions in this tissue [31].

## Materials and Methods

### Ethics statement

All studies and procedures involving animals were in strict accordance with the Harvard's Institutional Animal Care and Use Committee (IACUC), the Institutional Animal Care and Use Committee at Yale School of Medicine as well as French and European legislations for the “Protection of Vertebrate Animals used for Experimental and other Scientific Purposes” and were approved by the Departmental Direction of Populations Protection (approval number: B75-05-18). Husbandry, supply of animals as well as maintenance and care of the animals in EOPS (Exempt Of Pathogen Species) environments before and during experiments fully satisfies the animal's needs and welfare. Suffering to the animals has been kept to a minimum; no procedures inflicting pain have been performed.

### Generation of Notch(1–4)-CreERT2^SAT^ and Hes1-EmGFP^SAT^ constitutive Knock-in mice

#### Notch(1–4)-CreERT2^SAT^ constitutive Knock-in mice

Targeting vectors were based on 9.2 to 10.0 kb genomic fragments covering the promoter region (Notch1–4), exon 1 (Notch1–4), exon 2 (Notch1,3), exon 2–8 (Notch4) and surrounding sequences. The four individual genomic fragments obtained from a C57Bl/6J RP23 BAC library were modified in a similar fashion by replacing the exon 1 region between the translation inititation site and the end of exon 1 with a cassette containing the CreERT2 open reading frame, a polyadenylation signal and an FRT-flanked Neomycin resistance gene.

#### Hes1-EmGFP^SAT^ constitutive Knock-in mice

The targeting vector was based on a 10.2-kb genomic fragment from the Hes1 gene encompassing exons 1 to 4 and surrounding sequences. This fragment, obtained from a C57Bl/6J RP23 BAC library, was modified by replacing the genomic region from the translation initiation site in exon 1 to exon 3 with a cassette containing the EmGFP open reading frame, a polyadenylation signal and an FRT-flanked Neomycin resistance gene. A Thymidine Kinase (Tk) cassette was inserted at the 3′ end of the genomic fragment to allow for ganciclovir selection of ES cell clones displaying homologous recombination events.

#### ES cell culture

The quality tested C57BL/5NTac ES cell line was grown on a mitotically inactivated feeder layer comprised of mouse embryonic fibroblasts (MEF) in DMEM High Glucose medium containing 20% FBS (PAN Biotech GmbH) and 1200 u/mL Leukemia Inhibitory Factor (Millipore ESG 1107). 1×10^7^ cells and 30 µg of linearized DNA targeting vector were electroporated (Biorad Gene Pulser) at 240 V and 500 µF. Upon appropriate selection resistant colonies with a distinct morphology were isolated on day 9 after transfection and expanded in 96well plates. Correctly recombined ES cell clones were identified by Southern Blot analysis using external and internal probes and were frozen in liquid nitrogen.

#### Generation of mice

The animal study protocol was approved according to the German Animal Welfare Act by the local authority. Mice were kept in the animal facility at TaconicArtemis GmbH in microisolator cages (Tecniplast Sealsave). Feed and water were available *ad libitum*. Light cycles were on a 12∶12 h light∶dark cycle with the light phasing starting at 06∶00 h. Temperature and relative humidity were maintained between 21 and 23°C and 45 and 65%.

After administration of hormones, superovulated BALB/c females were mated with BALB/c males. Blastocysts were isolated from the uterus at dpc 3.5. For microinjection, blastocysts were placed in a drop of DMEM with 15% FCS under mineral oil. A flat tip, piezo actuated microinjection-pipette with an internal diameter of 12–15 micrometer was used to inject 10–15 targeted C57BL/6NTac ES cells into each blastocyst. After recovery, 8 injected blastocysts were transferred to each uterine horn of 2.5 days post coitum, pseudopregnant NMRI females. Chimerism was measured in chimeras (G0) by coat colour contribution of ES cells to the BALB/c host (black/white). Highly chimeric mice were bred to C57BL/6-Tg(CAG-Flpe)2Arte females mutant for the presence of the Flp recombinase gene. This allowed detection of germline transmission by the presence of black, strain C57BL/6, offspring (G1) and creation of selection marker deleted constitutive Knock-in mice by Flp-mediated removal, in one breeding step.

#### Genotyping of mice by PCR

Genomic DNA was extracted from tail tips using the NucleoSpin Tissue kit (Macherey-Nagel) and was analyzed by PCR in the presence of 2.0 mM MgCl2, 200 µM dNTPs, 100 nM of each primer, and 2 U of Taq DNA polymerase (Invitrogen) with the following primers detecting heterozygous or homozoygous Knock-in alleles:

Notch1: 1228_23: ATAGGAACTTCAAAATGTCGCG

1289_31: CACACTTCCAGCGTCTTTGG (312 bp KI)

Notch2: 1228_23: ATAGGAACTTCAAAATGTCGCG)

1270_23: CCCAACGGTGCCAAAAGAGC (513 bp KI)

Notch3: 1228_23: ATAGGAACTTCAAAATGTCGCG

1239_22: CCCAGCTGCTGCATCTCTGC (187 bp KI)

Notch4: 1228_23: ATAGGAACTTCAAAATGTCGCG

1273_28: GGCTTCTCCCTGGGCATGG (276 bp KI)

Hes1: 1487_35: CCCAAGTTCGGGTGAAGGC

1487_36: CCTTGGACAATGCCACCCAA (396 bp KI)

The Rosa26R and R26^mTmG^ reporter lines have been previously described [17], [22] and were obtained by the Jackson Laboratories.

### Histology, In Situ Hybridization, Immunofluorescence Labeling, and ß-Galactosidase Assay

Tissues were fixed and immunostained as previously described [31]. Immunofluorescence was performed on OCT-embedded frozen sections (5 µm), while immunohistochemistry on paraffin sections (5 µm) of intestines fixed in 4% paraformaldehyde (PFA) for 2 hours at room temperature. Where indicated, sections were counterstained with Hematoxylin and Eosin (Vector Laboratories), Nuclear Fast Red (Vector Laboratories) or PAS (Periodic Acid Schiff, VWR). For fluorescence staining, nuclei were stained with DAPI.

The primary antibodies used were: rabbit anti-lysozyme (cat# A0099, Dako Cytomation, 1/500), mouse anti-villin (made in our lab, 1/800), rabbit anti-chromogranin (cat# 20086, Immunostar, 1/200) and rabbit anti-mucin2 antibody (a generous gift of Dr Hansson, Goteborg University, 1/200). Identification of goblet cells on frozen sections was performed using Ulex Europeus Agglutinin (UEA) coupled to Cy3 (1/50) (Sigma). For fluorescence staining, nuclei were stained with DAPI. Cy3 or Alexa Fluor 633-coupled secondary antibodies (Invitrogen) were detected by compound epifluorescent and light microscopy or by an Apotome Microscope. Photomicrographic images were processed using Adobe Photoshop, with parallel images processed identically.

LacZ staining was carried out as described in Barker et al. [2]. In short, intestines were fixed for 2 hours on ice in 1% PFA, 0.2% glutaraldehyde, and 0.02% NP40 in PBS, washed twice in PBS, and subsequently stained overnight in 5 mmol/L K3FE(CN)_6_, 5 mmol/L K4FE(CN)_6_.3H2O, 2 mmol/L MgCl2, 0.02% NP40, and 1 mg/mL X-gal in PBS. After staining, tissues were washed twice in PBS and post-fixed overnight at 4°C in 4% PFA. LacZ-positive cells were counted on several stretches of proximal intestine totaling between 400 and 1000 crypts. The number of tracing events was counted at the most proximal part of intestines at different time points. At least 2 mice per time point were analyzed. Other organs were snap-frozen in OCT without fixation. Frozen sections were then fixed in 0.7% PFA, 0.05% glutaraldehyde, and 0.02% NP40 in PBS at room temperature for 15 minutes, washed twice in PBS and incubated in X-gal solution as described above.

For in situ hybridization, the proximal small intestine was dissected and post-fixed overnight by immersion in 4% PFA, then cryoprotected in 30% sucrose in 4% PFA and sectioned in the transverse plane on a Leica sledge cryomicrotome at 40 µm (Leica Microsystems, Germany). Sections were then mounted on slides and processed for non-radioactive in situ hybridization as described previously with minor modifications [40]. RNA probes complementary to mouse *Notch1* and *Notch2* cDNA were prepared and labeled with digoxigenin-11-UTP. Sections were analyzed using a Zeiss AxioImager fitted with a Zeiss AxioCam MRc5 digital camera. Images were captured using AxioVision AC software (Zeiss) and assembled using Adobe Photoshop.

#### Lineage Analysis and Notch inhibition

For lineage analysis in the intestine, four week-old mice, including control mice, received one single intra-peritoneal injection of tamoxifen (ICN) (50 or 5 mg/Kg of mouse body weight) and were analyzed at the indicated chase time points. To confirm the absence of leakiness of the new transgenic lines, we analyzed Notch(1–2)-CreERT2^SAT^/+; R26R/+ and N(1–2)-CreERT2^SAT^/+; R26^mTmG^/+ mice without tamoxifen administration and found no LacZ nor GFP staining in these control mice. For expression analysis in other organs we used a single intra-peritoneal injection of 4-Hydroxytamoxifen (4-OHT) (50 mg/kg of mouse body weight).

Pharmacological inhibition of Notch signaling in Hes1-EmGFP^SAT^ mice was achieved by intraperitoneal administration of the γ-secretase inhibitor dibenzazepine (DBZ: 20 µM/kg body weight in 0.5% HPMC, 0.1% w/v NP40 in water; Calbiochem). Adult mice were injected with DBZ for 4 consecutive days and analyzed 3–4 days after the last injection.

#### Evaluation of stem cell replacement rate

To fit the data for monoclonal conversion (Fig. 3E), we used the equations associated with the neutral drift theory [25], [30]. In our case, however, we do not include any transit time, since we are counting cells directly from crypts longitudinal sections. We assumed that there are 16 stem cells/crypt, and that what we call a fully labeled crypt has at least 14 labeled stem cells; this arbitrary cutoff affects the results only marginally. The stem cell renewal rate, which defines the only relevant time for monoclonal conversion, was then extracted, along with the standard error, using the NonLinearModelFit function in Mathematica: λ = 1.32±0.05/days (P<10^−5^) [25], [30]. Using this parameter, we generated the expected theoretical curve for the fraction of marked crypts in time (Figure 3F).

## Supporting Information

Figure S1 **Targeting of Notch(1–4) loci and expression of the four Notch receptor paralogues.** A) Schematic representation of the knock-in strategy. The targeting allele contains the *CreERT2* gene and a polyadenylation signal (pA) placed in frame with the start codon of each Notch gene, replacing much of the ORF. In each of the four N(1–4)-CreERT2^SAT^ knock-in (KI) strains, one allele of the targeted locus expresses the CreERT2 fusion protein under the control of the corresponding Notch paralogue promoter. Blue boxes represent exons. B–K) Frozen sections labeled by X-gal (blue) showing the expression pattern of N1-CreERT2^SAT^/+; R26R/+ (B,F), N2-CreERT2^SAT^/+;R26R/+ (C,G), N3-CreERT2^SAT^/+;R26R/+ (D,H,J) and N4-CreERT2^SAT^/+;R26R/+ (E,I,K) in adult mouse liver (B–E), heart (F–I,K) and brain (J) 24 h after 4-OHT administration. Notch2 is the most abundant paralogue in the liver (C). Notch3 is expressed in mural cells of small and medium size penetrating arteries in the brain (J,J′). Notch4 is expressed in endothelial cells of the heart (K). Counterstain: Nuclear Fast Red (B–I) eosin (K). Scale bars: 50 µm in B–J, 25 µm in K.
(TIF)

Figure S2 **Notch1 expression in the mouse intestine present a descending gradient.** X-gal stained whole mounts segments of duodenum (A), jejunum (B), ileum (C) and colon (D) of N1-CreERT2^SAT^/+;R26R/+ mice 21 days after tamoxifen administration shows a drastic decrease in labeled crypt-villus units in the distal parts of the intestine. Scale bar: 1 mm.
(TIF)
