# Supplementary figures and images for: Notch Lineages and Activity in Intestinal Stem Cells Determined by a New Set of Knock-In Mice
Source: PLoS One. 2011 Oct 3;6(10):e25785. doi: 10.1371/journal.pone.0025785 (PMC3185035; doi:10.1371/journal.pone.0025785)

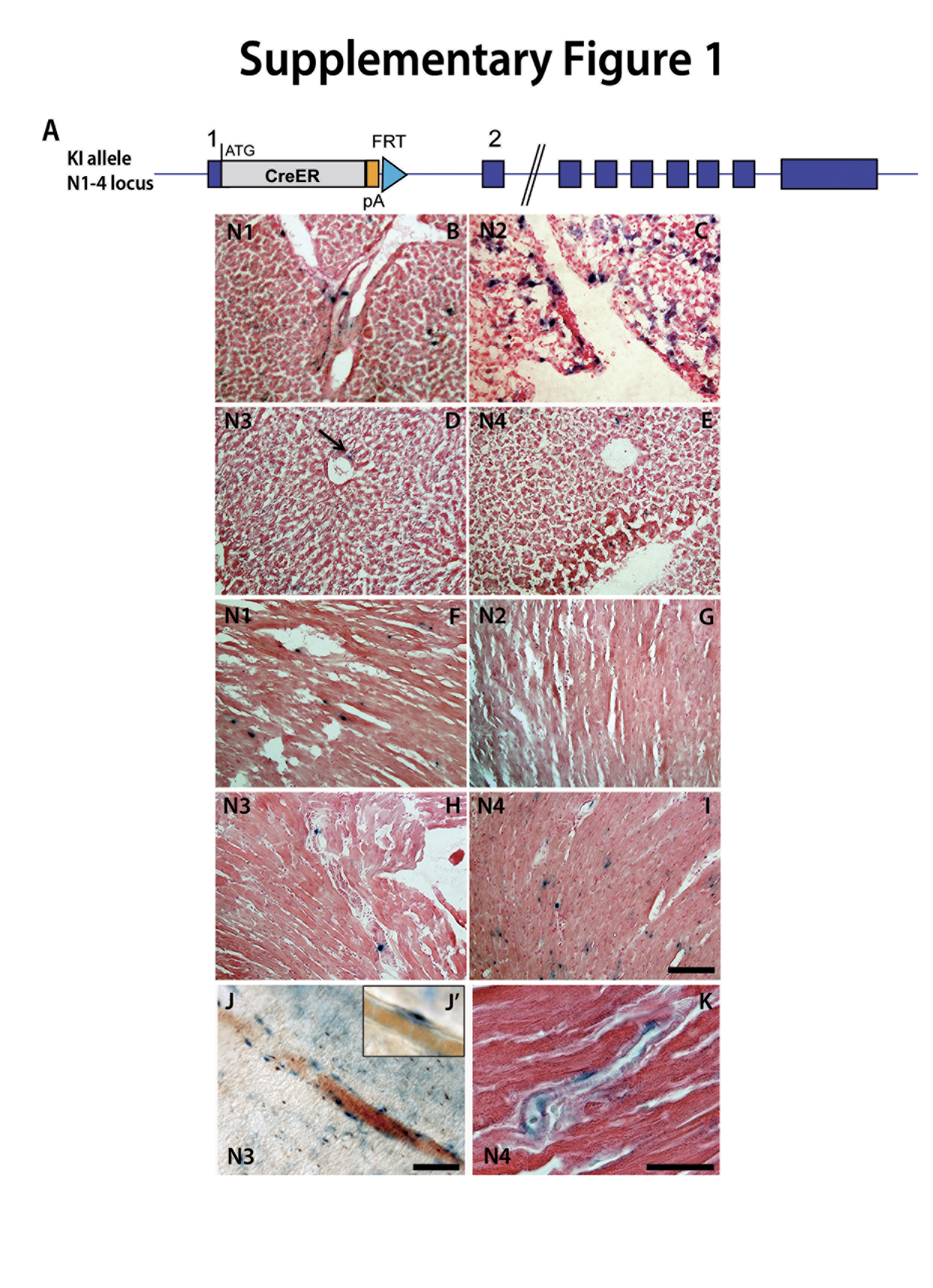

Supplement: Figure S1 — Targeting of Notch(1–4) loci and expression of the four Notch receptor paralogues. A) Schematic representation of the knock-in strategy. The targeting allele contains the CreERT2 gene and a polyadenylation signal (pA) placed in frame with the start codon of each Notch gene, replacing much of the ORF. In each of the four N(1–4)-CreERT2SAT knock-in (KI) strains, one allele of the targeted locus expresses the CreERT2 fusion protein under the control of the corresponding Notch paralogue promoter. Blue boxes represent exons. B–K) Frozen sections labeled by X-gal (blue) showing the expression pattern of N1-CreERT2SAT/+; R26R/+ (B,F), N2-CreERT2SAT/+;R26R/+ (C,G), N3-CreERT2SAT/+;R26R/+ (D,H,J) and N4-CreERT2SAT/+;R26R/+ (E,I,K) in adult mouse liver (B–E), heart (F–I,K) and brain (J) 24 h after 4-OHT administration. Notch2 is the most abundant paralogue in the liver (C). Notch3 is expressed in mural cells of small and medium size penetrating arteries in the brain (J,J′). Notch4 is expressed in endothelial cells of the heart (K). Counterstain: Nuclear Fast Red (B–I) eosin (K). Scale bars: 50 µm in B–J, 25 µm in K. (TIF) [file pone.0025785.s001.tif]

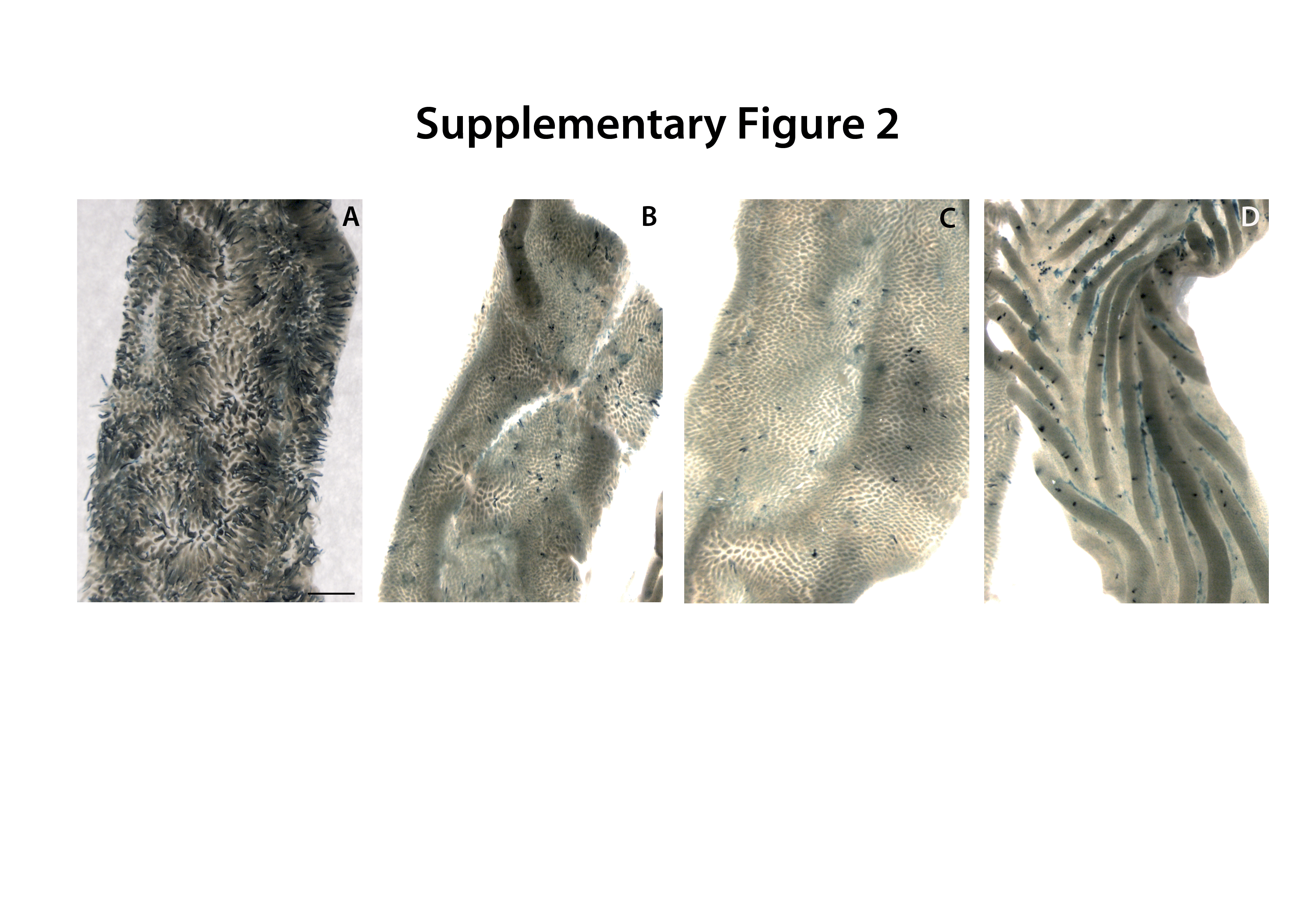

Supplement: Figure S2 — Notch1 expression in the mouse intestine present a descending gradient. X-gal stained whole mounts segments of duodenum (A), jejunum (B), ileum (C) and colon (D) of N1-CreERT2SAT/+;R26R/+ mice 21 days after tamoxifen administration shows a drastic decrease in labeled crypt-villus units in the distal parts of the intestine. Scale bar: 1 mm. (TIF) [file pone.0025785.s002.tif]
